# Identification and evolutionary analysis of the metal‐tolerance protein family in eight Cucurbitaceae species

**DOI:** 10.1002/tpg2.20167

**Published:** 2021-11-06

**Authors:** Xinchen Jiang, Junliang Yin, Lixin Wang, Keyong Xi, Xiongmeng Zhu, Gang Li, Yongxing Zhu, Yiqing Liu

**Affiliations:** ^1^ Hubei Key Laboratory of Waterlogging Disaster and Agricultural Use of Wetland/College of Horticulture and Gardening Yangtze Univ. Jingzhou Hubei 434000 China; ^2^ College of Horticulture Hebei Agricultural Univ. Baoding Hebei 071001 China

## Abstract

Metal‐tolerance proteins (MTPs) are divalent cation transporters and play fundamental roles in plant metal tolerance and ion homeostasis. Despite that, a systematic investigation of MTPs in Cucurbitacea is still lacking. In this study, 142 MTPs were identified from 11 released genomes of 8 Cucurbitaceae species. They were phylogenetically separated into three clusters (Zn‐cation diffusion facilitator proteins [CDFs], Fe/Zn‐CDFs, and Mn‐CDFs) and further subdivided into seven groups (G1, G5, G6, G7, G8, G9, and G12). Characterization analysis revealed that most MTPs were plasma membrane‐located hydrophobic proteins. Motif and exon/intron analysis showed that members in the same group contained similar conserved motifs and gene structures. Moreover, 98 pairs of segmental‐like duplication events were found. The nonsynonymous/synonymous substitution ratios between each pair were less than 1, implying that Cucurbitaceae MTPs were under purification selection. Expression profiling suggested that several MTP genes, such as *CsCLMTP1*, *CmeMTP3*, *LsMTP3*, and *Cl97103MTP3*, were constitutively expressed in corresponding Cucurbitaceae species, and their expression levels were not significantly altered by NaCl, drought, or pathogen infection. The expression patterns of cucumber MTP genes under Zn^2+^, Cu^2+^, Mn^2+^, and Cd^2+^ stress were studied by quantitative real‐time polymerase chain reaction and the results showed that these MTPs were induced by at least one metal ion, suggesting their involvement in metal tolerance or transportation. The identification and comprehensive investigation of MTP family members will provide a basis for the analysis of ion transport functions and ion tolerance mechanisms of Cucurbitaceae MTPs.

## Core Ideas

- A total of 142 metal‐tolerance proteins (MTPs) were identified in eight Cucurbitaceae species.
- Cucurbitaceae MTPs were under strong purification selection.
- Cucurbitaceae MTPs showed tissues and treatment‐specific expression patterns.
- Cucumber MTPs were induced by metal ions.

**Abbreviations**
aa: amino acid
CAX: cation exchanger
CDF: cation diffusion facilitator proteins
FPKM: fragments per kilobase of transcript per million fragments mapped
GRAVY: grand average of hydropathy
Ka: nonsynonymous substitution
Ks: synonymous substitution
MTPs: metal‐tolerance proteins
NRAMP: natural resistance‐associated macrophage proteins
qPCR: quantitative polymerase chain reaction
TMDs: transmembrane domains.

## 1 INTRODUCTION

Heavy metal ions, such as Cu^2+^, Fe^3+^, Zn^2+^, Co^3+^, Ni^2+^, and Mn^2+^, could be classified into essential and nonessential micronutrients, which play important roles in plant growth and development (Maleki et al., 2017). However, with the development of agriculture, the excessive usage of agricultural amendments induces tremendous heavy metal pollutants into the environment (Zhu et al., 2019a). For example, the proper application of essential micronutrients is necessary and beneficial for normal plant growth, but excessive micronutrient accumulation in the soil could trigger toxic effects. In addition, a lower concentration of nonessential metal ions, such as Cd^2+^ and Hg^2+^, could be toxic to organisms because of the formation of reactive oxygen species and/or competition with other essential ions (Wu et al., 2019; Yuan et al., 2012). Thus, heavy metal stress negatively and severely affects plant growth. The absorption of heavy metals in edible parts of crops, fruits, and vegetables seriously affects human health (Ghori et al., 2019). To maintain metal homeostasis at the cellular level, plants have evolved a complex network to respond to heavy metal stress through uptake, extrusion, chelation, and translocation mechanisms (Bhat et al., 2019; Wu et al., 2013). Among them, several groups of cation transporters, including the cation exchanger (CAX) family, natural resistance‐associated macrophage proteins (NRAMP), and cation diffusion facilitator proteins (CDF), have been widely studied and have been proven to play vital roles in this biological process.

In the plant kingdom, the CDF family is usually designated as metal‐tolerance proteins (MTPs) (Lang, et al., 2011; Ricachenevsky et al, 2013). Plant MTPs are divalent‐cation/H^+^ antiporters and play important roles in metal efflux from the cytoplasm; thus, they provide a fundamental contribution to the metal tolerance and homeostasis of plants (Fu et al., 2017). Previously, systemic identification and phylogenetic analysis was performed to comprehensively investigate plant MTPs, and the results showed that, based on their proposed and confirmed substrate specificity, MTPs could be classified into three major clusters: Mn‐CDFs, Fe/Zn‐CDFs, and Zn‐CDFs (Montanini et al., 2007). Furthermore, according to phylogenetic analysis and annotation performed on *Arabidopsis*, MTPs could be further divided into seven primary groups (Gustin et al., 2011). Following this classification, plant Zn‐CDFs comprise G1 (Group 1), G5, and G12; the Fe/Zn‐CDF cluster comprises G6 and G7; and G8 and G9 belong to the Mn‐CDFs (Gustin et al., 2011). Initially, 12 and 10 MTP genes were identified in model plants *Arabidopsis thaliana* and rice (*Oryza sativa* L.) (Montanini et al., 2007), respectively, and since then, the function of several MTPs has been explored. For instance, AtMTP5 together with AtMTP12 could transport Zn^2+^ into the Golgi apparatus in Arabidopsis (Fujiwara et al., 2015). In tea plants (*Camellia sinensis* L.), heterologous expression of plasma membrane localized CsMTP8.2 in Arabidopsis revealed that it is a Mn^2+^‐specific transporter that contributes to the efflux of excess Mn^2+^ from plant cells (Zhang et al., 2020). In *Populus trichocarpa* Torr. & A. Gray ex Hook, PtrMTP8.1, PtrMTP9, and PtrMTP10.4 could transport Mn^2+^ in yeast cells, and PtrMTP6 showed the ability to transport Co^2+^, Fe^2+^, and Mn^2+^ (Gao et al., 2020). This research provides a systematic understanding of the subcellular localization of MTPs and their functions in metal ions transportation and lay a foundation for MTP gene function analysis for other species.

**Core Ideas**

- A total of 142 metal‐tolerance proteins (MTPs) were identified in eight Cucurbitaceae species.
- Cucurbitaceae MTPs were under strong purification selection.
- Cucurbitaceae MTPs showed tissues and treatment‐specific expression patterns
- Cucumber MTPs were induced by metal ions.

Cucumber (*Cucumis sativus* L.), belonging to *Cucumis*, is one of the most widely cultivated vegetable crops in the world (Zhu et al., 2020). In cucumber, four MTP genes have been investigated. For example, *CsMTP1* and *CsMTP4* are involved in Zn^2+^ homeostasis and Cd^2+^ sequestration in cucumber cells (Migocka, Kosieradzka et al., 2015). *CsMTP8* is an Mn^2+^ transporter localized in the vacuolar membrane, which is highly and specifically expressed in cucumber roots and was markedly upregulated or downregulated under elevated Mn^2+^ or Mn^2+^ deficiency, respectively (Migocka et al., 2014). *CsMTP9*, which functions as an H^+^‐coupled antiporter, could transport Mn^2+^ and Cd^2+^ from cucumber root cells (Migocka, Papierniak et al., 2015). These results largely expanded our understanding of cucumber MTP genes. However, a systemic analysis of MTP at the Cucurbitaceae level is still missing, and the detailed roles of most Cucurbitaceae MTP genes have not been comprehensively studied.

The Cucurbitaceae family contains about 1,000 species in 130 genera, such as the typical *Trichosanthes*, *Lagenaria*, *Luffa*, *Benincasa*, *Momordica*, *Cucumis*, *Citrullus*, *Cucurbita*, *Bryonopsis*, and *Corallocarpus* (Renner & Schaefer, 2016). Cucurbits form a large and important group of vegetable crops cultivated extensively in subtropical and tropical countries and have long been of economic significance (Rajasree et al., 2016). In China, various types of heavy metal pollution have been reported in vegetable soil. Although heavy metal pollution (mainly Cd, Zn, Cu, and Mn) is mainly moderate to light in China, the polluted area has been expanding (Feng et al., 2018). Considering the important function of MTPs, the identification and comprehensive investigation of MTP family members will provide a basis for the analysis of ion transport functions and ion tolerance mechanisms of Cucurbitaceae MTPs. Currently, whole‐genome sequencing of several Cucurbitaceae species has been completed, which allowed the MTP family genes to be mined by employing various bioinformatics tools and approaches.

To systematically explore MTPs in the Cucurbitaceae family and their structure and expression patterns, MTP gene family identification was performed in eight Cucurbitaceae species. In addition, analyses including phylogenetic relationships, gene structures, protein motifs, and expression patterns in response to heavy metal stress were performed. The identification and comprehensive investigation of MTP family members in Cucurbitaceae will provide us with an opportunity to understand transport proteins both in terms of their structural characteristics and in terms of their physiological roles.

## 2 MATERIALS AND METHODS

### 2.1 Identification of the MTP gene family in sequenced Cucurbitaceae species

The protein sequences of *Arabidopsis* MTPs (http://www.arabidopsis.org/index.jsp) (Montanini et al., 2007) were used as queries to conduct BLASTp searches against accessible genomes of Cucurbitaceae species with a cutoff e‐value of 1e^−10^ (http://cucurbitgenomics.org/). The keyword ‘metal tolerance protein’ was used to search the Cucurbitaceae genomes. The retrieved sequences were submitted to InterProScan (http://www.ebi.ac.uk/interpro/) to verify the MTP domains (IPR002524). All of the protein sequences that contained the typical MTP domain were considered potential candidates.

### 2.2 Characterization of gene structures and protein transmembrane structures

Eleven genome sequences and gff3 files belonging to eight Cucurbitaceae species, namely, *Cucumis sativus* (‘Chinese Long’, ‘Gy14’, ‘PI183967’), *Citrullus lanatus* (‘97103’, ‘Charleston Gray’), *Cucumis melo*, *Cucurbita argyrosperma*, *Cucurbita maxima*, *Cucurbita moschata*, *Cucurbita pepo*, and *Lagenaria siceraria*, were downloaded from the Cucurbit Genomics Database (http://cucurbitgenomics.org/). The chromosomal location and exon/intron structures of MTP genes were extracted from the genome gff3 annotation files. These MTPs were anchored according to the gene position information using MapInspect software (http://mapinspect.software.informer.com) (Voorrips, 2002). The Gene Structure Display Server (http://gsds.cbi.pku.edu.cn/) was exploited to determine the exon/intron structures of individual MTPs by aligning the cDNA sequences to their corresponding genomic DNA sequences (Guo et al., 2007). The online tool ProtParam (https://web.expasy.org/protparam/) was used to predict features of MTP proteins, including the isoelectric point, relative molecular weight, instability index, atomic composition, and amino acid composition. Subcellular localizations were predicted using the WoLF PSORT tool. which is available at http://www.genscript.com/psort/wolf_psort.html. The putative transmembrane regions were predicted using the TMHMM Server V.2.0 (http://www.cbs.dtu.dk/services/TMHMM/). The conserved motifs in MTPs were identified using the MEME motif search tool (http://memesuite.org/tools/meme) (Bailey et al., 2009). Default parameters were used in this study, except that the maximum number of motifs was set to five. The motif patterns were drawn by TBtools software (https://github.com/CJ‐Chen/TBtools).

### 2.3 Multiple protein sequence alignment and phylogenetic analysis of MTPs

ClustalW2 (v2.1, http://www.clustal.org/) was used for the sequence alignment of Cucurbitaceae MTPs. An unrooted phylogenetic tree was constructed by using MEGA7 (http://www.megasofware.net/) with the neighbor‐joining method based on the LG model, and 1,000 bootstrap test replicates were used during tree construction (Tamura et al., 2013). The phylogenetic tree was illustrated using Interactive Tree of Life (IToL, v3.2.317; http://itol.embl.de). The combined tree was generated to systematically classify MTPs and show their phylogenetic relationships.

### 2.4 Synteny analysis

Synteny analysis of MTP genes among 11 Cucurbitaceae genomes was performed. The orthologs of MTP genes between each two genomes were analyzed by reciprocal blast with e‐value 10^−5^. According to the reciprocal blast output, duplication events were identified by using the McScanX software (Wang et al., 2012).

### 2.5 Calculation of nonsynonymous (Ka) and synonymous (Ks) substitutions and Ka/Ks ratios

The Ks and Ka rates (were calculated by using the DnaSP5.0 software) (Librado & Rozas, 2009).

### 2.6 RNA‐seq data analysis

To explore the expression patterns of MTP genes, the expression levels represented by FPKM (fragments per kilobase of transcript per million fragments mapped) values were collected from the Cucurbit Genomics Database (http://cucurbitgenomics.org/). The expression profiles were visualized using R package “pheatmap” (Yin et al., 2020).

### 2.7 Plant growth and stress treatments

Cucumber seeds (*Cucumis sativus* L. ‘JinYou 1’; Xintiandi Co.) were sterilized in a water bath (55 °C) for 15 min and then germinated in the dark on moist filter paper in Petri dishes at 28 °C. Germinated seeds were sown in a commercial mixed substrate and grown in a greenhouse at Yangtze University with natural sunlight (25 °C/18 °C, 50–60% relative humidity). Two‐leaf seedlings were transplanted into 30‐L plastic pots filled with 1/2 strength modified Hoagland nutrient solution (Hoagland & Arnon, 1950). For the Zn, Mn, Cu, and Cd treatments, 5 d after transplanting, the seedlings were transferred into new hydroponic solutions containing 100 μM ZnSO_4_•7H_2_O, 100 μM CdCl_2_, 400 μM MnSO_4_•H_2_O, or 40 μM CuSO_4_•5H_2_O, respectively. After 3 d of treatment, the root and leaf samples of each treatment were harvested separately, and then stored at –80 °C for RNA isolation.

#### 2.7.1 RNA extraction, cDNA synthesis, and quantitative real‐time polymerase chain reaction

Total RNA was isolated from 0.2‐g samples using TRIzOL reagent (Invitrogen) following the manufacturer's instructions. The first‐strand cDNA for quantitative polymerase chain reaction (qPCR) analysis was synthesized from 500 ng of total RNA using HiScript Reverse Transcriptase (Vazyme) based on the manufacturer's instructions, including a special step for genomic DNA digestion. The qPCR experiments were conducted on a CFX 96 Real‐Time PCR system (Bio‐Rad) using Cham SYBR qPCR Master Mix (Vazyme) with specific primers (Supplemental Table S1). The relative quantity was calculated using the 2^–△△Ct^ method (Yin et al., 2018). Each treatment included three biological replications, and each replication included two technical replications.

## 3 RESULTS AND DISCUSSION

### 3.1 MTPs were identified from sequenced Cucurbitaceae species

Heavy metals, such as Fe, Mn, Cu, Cd, and Zn, have long accumulated in soils through industrial waste and sewage disposal. In plants, MTPs play an important role in transporting metal ions from the cytosol either by sequestration into vacuoles or by export to extracellular compartments to avoid cellular damage. Although MTP genes have been studied in several plants, such as *Arabidopsis* (Montanini et al., 2007), wheat (*Triticum aestivum* L.) (Vatansever et al., 2017), sweet orange [*Citrus sinensis* (L.) Osbeck] (Fu et al., 2017), and turnip (*Brassica rapa* var. *rapa*) (Li et al., 2018), comprehensive molecular evolutionary study remains lacking in Cucurbitaceae. Cucurbitaceae plants have long been considered to be of biological and economic importance (Yang & Walters, 1992; Zhu et al., 2019b). In the past few years, 11 genomes belonging to eight Cucurbitaceae species have been sequenced, which allowed MTP family genes to be mined and systemically analyzed in Cucurbitaceae. To explore the characteristics, evolutionary processes, and distinctive biological functions of MTPs, MTP family genes were identified from 11 genomes and used to perform comprehensive analyses. In total, 142 MTPs were identified, including 10, 13, and 14 in *Cucumis sativus* Chinese Long, Gy14, and PI183967 genomes, respectively; 10 and 10 in *Citrullus lanatusv* 97103 and Charleston Gray, respectively; nine in *Cucumis melo*; 16 in *Cucurbita maxima*; 17 in *Cucurbita moschata*; 18 in *Cucurbita pepo*; 16 in *Cucurbita argyrosperma*; and nine in *Lagenaria siceraria* (Table 1, Supplemental Table S2). The chromosome distribution of MTPs was determined in each Cucurbitaceae genome. In *Citrullus lanatus* (97103), three, two, one, two, and two Cl97103MTP genes were found on Chr2 (chromosome 2), Chr3, Chr6, Chr9, and Chr10, respectively. In *Citrullus lanatus* (Charleston Gray), two, two, one, one, one, and two ClCGMTP genes were found on Chr2, Chr3, Chr5, Chr6, Chr9, and Chr10, respectively. In *Cucurbita maxima*, Chr1, Chr6, Chr8, Chr10, Chr11, and Chr20 contained one CmaMTP gene each; Chr2 and Chr4 contained 2 CmaMTP genes each, and Chr14 and Chr17 contained 3 CmaMTP genes each. In *Cucurbita moschata*, Chr6, Chr8, Chr10, Chr11 and Chr20 contained one CmoMTP genes, respectively; Chr1, Chr2, and Chr4 contained two CmoMTP genes; Chr14 and Chr17 contained three MTP genes. In *Cucurbita pepo*, Chr1, Chr2, Chr3, Chr5 and Chr12 contained two CpMTP genes; Chr4, Chr11, Chr16, Chr17, and Chr18 contained one CpMTP each. In *Cucumis melo*, Chr0 (unanchored scaffolds) contained five CmeMTP genes and Chr4 contained four CmeMTPs. In *C*. *sativus* Chinese Long, five chromosomes contained CsCLMTP genes except Chr2 and Chr4. In *C*. *sativus* cv. Gy14, CsGyMTPs were distributed on all chromosomes except Chr4. In *C*. *sativus* cv. PI183967, three, four, two, two, and three CsPIMTPs were distributed on Chr1, Chr 3, Chr 5, Chr 6, and Chr 7 (Supplemental Table S2).

**TABLE 1:** Characteristics of the identified metal‐tolerance proteins genes in Cucurbitaceae species

**Species**	**Zn‐CDF**	**Fe/Zn‐CDF**	**Mn‐CDF**	**Total**
*Cucurbita argyrosperma*	2	9	5	16
*Citrullus lanatus* cv. 97103	2	4	4	10
*Citrullus lanatus* cv. Charleston gray	2	5	3	10
*Cucurbita maxima*	3	8	5	16
*Cucumis melo*	2	4	3	9
*Cucurbita moschata*	3	9	5	17
*Cucurbita pepo*	5	8	5	18
*Cucumis sativus* cv. Chinese long	2	5	3	10
*Cucumis sativus* cv. Gy14	2	6	5	13
*Cucumis sativus* cv. PI183967	2	7	4	14
*Lagenaria siceraria*	3	2	4	9

*Note*. CDF, cation diffusion facilitator protein.

Protein family searches demonstrated that the identified Cucurbitaceae MTPs belonged to the cation efflux (CDF) family (Supplemental Table S2). Further analysis revealed that the physiological and biochemical features of these MTPs vary. The length of each MTP ranges from 74 amino acids (aa) (CsGyMTP12;1) to 3,117 aa (CmaMTP8), most of which were within 350 to 550 aa; the average aa length was similar with around 450 aa among 8 species. The molecular weight of MTPs ranged from 8.96 (CsGyMTP12;1) to 353.50 (CmaMTP8) kDa, with an average of 53.3 kDa. These findings indicated that Cucurbitaceae MTPs were comparatively small and have similar molecular size. The isoelectric point (pI) values, an important physicochemical property of proteins, ranged from 4.69 (CpMTP7;1) to 9.49 (CsPIMTP12;1). Moreover, the pI values of most MTPs in Zn‐CDFs and Mn‐CDFs clusters were below 7.0, whereas members belonging to Fe/Zn‐CDFs were higher than 7.0, implying that Cucurbitaceae Zn‐CDFs and Mn‐CDFs were usually acidic, and Fe/Zn‐CDFs were basic. In addition, the grand average of hydropathy (GRAVY) values varied from −1.5 (CaMTP9;1) to 0.658 (CaMTP5;3), with an average value of 0.0511, showing that Cucurbitaceae MTPs generally had weak hydrophilicity. Consistent with their membrane location and divalent cation transporting function, most MTPs contained —four to six typical transmembrane regions, except CmaMTP8, which contained 23 as the highest, and CaMTP9;2, CmeMTP9, CmoMTP12;3, CsGyMTP12;1, CsGyMTP8;2, and CsPIMTP8, which contained 0 as the least (Supplemental Table S2). Previous studies have shown the different subcellular localizations of MTPs. For instance, the MTPs could be at the cell membrane in bacteria, vacuolar membrane in yeast or plants, and Golgi apparatus in mammals or plants (Haney et al., 2005; Peiter et al., 2007). In cucumber, CsMTP1 and CsMTP4 had been indicated mainly localization at the tonoplast, and CsMTP9 was proved to be a root‐specific plasma membrane transporter, playing an important role in metal transport (Migocka, Kosieradzka et al., 2015; Migocka, Papierniak et al., 2015). In this study, predication analysis showed that the subcellular localization of most MTPs was at the plasma membrane, but some individual ones were located at the nucleus, endoplasmic reticulum, vacuoles, chloroplast, and cytoplasm, implying that certain MTPs could transport heavy metals into subcellular compartments (Supplemental Table S2).

### 3.2 Evolutionary relationships among Cucurbitaceae MTPs were determined by phylogenetic analysis

**FIGURE 1:**
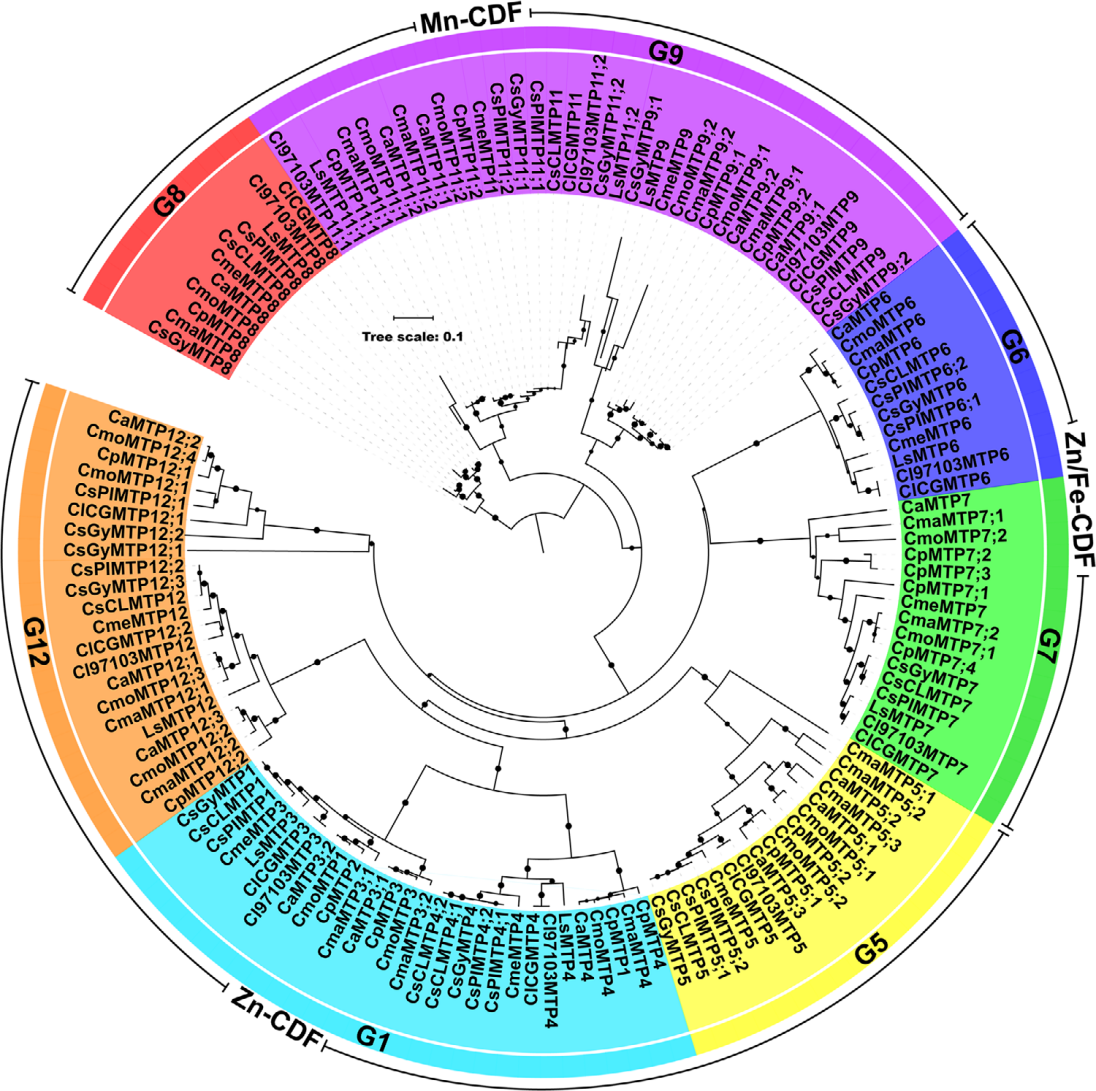
Phylogenetic tree of metal‐tolerance protein (MTP) proteins from *Cucumis sativus*, *Citrullus lanatus*, *Cucumis melo*, *Cucurbita argyrosperma*, *Cucurbita maxima*, *Cucurbita moschata*, *Cucurbita pepo*, and *Lagenaria siceraria*. The tree was generated with MEGA7 software using the neighbor‐joining method. The proteins were divided into three groups (Zn‐MTPs, Fe/Zn‐MTPs, and Mn‐MTPs) according to their phylogenetic relationships and previously reported classification methods, and seven groups, namely, G1, G5, G6, G7, G8, G9, and G12 (light blue: G1; yellow: G5; dark blue: G6; green: G7; red: G8; purple: G9; orange: G12)

MTPs are important metal transporters (Fu et al., 2017). Comparison of multisequence alignment and phylogenetic analysis could provide functional prediction for undefined genes (Zhu et al., 2019c). To gain insights into the evolutionary relationships of the MTPs among Cucurbitaceae species, 142 MTP protein sequences were used to build a phylogenetic tree. As shown in Figure 1, the phylogenetic relationships indicated that all of the MTPs could be classified into three clusters (namely, Zn‐CDFs, Fe/Zn‐CDFs, and Mn‐CDFs), which could be further separated into seven groups (G1, G5, G6, 7G, G8, G9, and G12). Each of the eight studied Cucurbitaceae species contributed at least one member of the MTP genes to each group. MTP1, 2, 3, and 4, MTP5, and MTP12 belong to the G1, G5, and G12 groups, respectively, which are Zn‐CDFs. MTP6 and 7, and MTP8, 9, and 11 belong to Zn/Fe‐CDFs and Mn‐CDFs, respectively. Group 9 contained the largest number of MTP genes (35), whereas G8 contained the smallest number of MTP genes (11) (Figure 1). According to the principle of phylogenetic tree, genes in the same cluster, even in different species, generally have a similar structure and function (Zhu et al., 2019c). To better understand the evolutionary relationships of MTPs among Cucurbitaceae, Arabidopsis, maize (*Zea mays* L.), and rice, a phylogenetic tree was constructed based on their protein sequences (Supplemental Figure S1). The results showed that orthologs of most AtMTPs were found in Cucurbitaceae and other species. Using cucumber as an example, multiple genes homologous to AtMTP1, AtMTP4, AtMTP5, AtMTP6, AtMTP7, AtMTP8, AtMTP9, AtMTP11, and AtMTP12 were identified in cucumber (Supplemental Figure S1). However, no genes homologous to AtMTP2, AtMTP3, or AtMTP10 were found in Cucurbitaceae. Furthermore, homologous genes of AtMTP2 and AtMTP10 were also not identified in monocotyledon maize and rice, suggesting that homologous genes in Cucurbitaceae, maize, and rice may have been lost during the evolutionary process or due to a dependent gene divergence event occurring in Arabidopsis. However, the incomplete genome factor should also be considered. Specifically, orthologous AtMTP8 was identified in the eight dicotyledonous Cucurbitaceous species and monocotyledon maize and rice, indicating evolutionary conservation of MTP8. AtMTP8 is a Mn^2+^ transporter located in the vacuolar membrane that determines the tolerance to iron deficiency and the Mn^2+^ and Fe^2+^ distribution in seeds (Chu et al., 2017). In tea plants, heterologous expression of *CsMTP8*.*2* in Arabidopsis revealed that CsMTP8.2, which is localization in the plasma membrane, contributes to the efflux of excess Mn^2+^ from plant cells. In cucumber, vacuolar membrane‐located CsMTP8 is specifically expressed in cucumber roots and was stimulated under Mn^2+^ excess or Mn^2+^ deficiency (Migocka et al., 2014), whereas OsMTP8 was the most abundant in the shoots of rice and function to sequestrate excess Mn^2+^ into vacuoles in rice and is required for Mn^2+^ tolerance in shoots (Chen et al., 2013). MTP8 in other Cucurbitaceae species may be functionally similar to CsMTP8 and AtMTP8 regarding its role in plant response to Mn^2+^ toxicity. Nevertheless, molecular and physiological experimental characterization is still required to confirm their function. These results suggested that most Cucurbitaceae MTPs were evolutionary conserved and, thus, the known functions of homology genes in other plants could provide important clues for the function analysis in Cucurbitaceae species.

### 3.3 Gene structure and conserved domain analysis of MTPs in cucurbitaceae

To further investigate the function and structural features of MTPs from the evolutionary level, the protein sequences, conserved motifs and domains, and contents of exons and introns were evaluated by ClustalW, MEME and GSDS. The fourth, fifth, and sixth transmembrane domains (TMDs) were conserved among MTPs. The most conserved regions were the amphipathic TMDs I, II, V, and VI, which are likely involved in metal transfer (Haney et al., 2005). The Mn‐CDF sequences could be differentiated by the consensus sequence DxxxD (D = Asparticacid; x = any amino acid) in TMD V, which appeared as HxxxD (H = Histidine; x = any amino acid) in all other CDF sequences from the Zn‐ and Zn/Fe‐CDFs, with D being the highly conserved aspartate residue important for CDF function (Montanini et al., 2007). Thus, residues that could represent candidate sites of functional divergence of the CDF groups were further investigated. In this study, the conserved sequence HxxxD was identified in four Cl9710MTP, four CmeMTP, five CsCLMTP, two CsCyMTP (two CsPIMTP, three LsMTP, nine CmaMTP, eight CmoMTP, nine CpMTP, and seven CaMTP proteins). These MTP members belong to the Zn‐MTP group, whereas the motif DxxxD was observed in the Mn‐MTPs subgroup, including MTP8, 9, and 11 (File S1).

**FIGURE 2:**
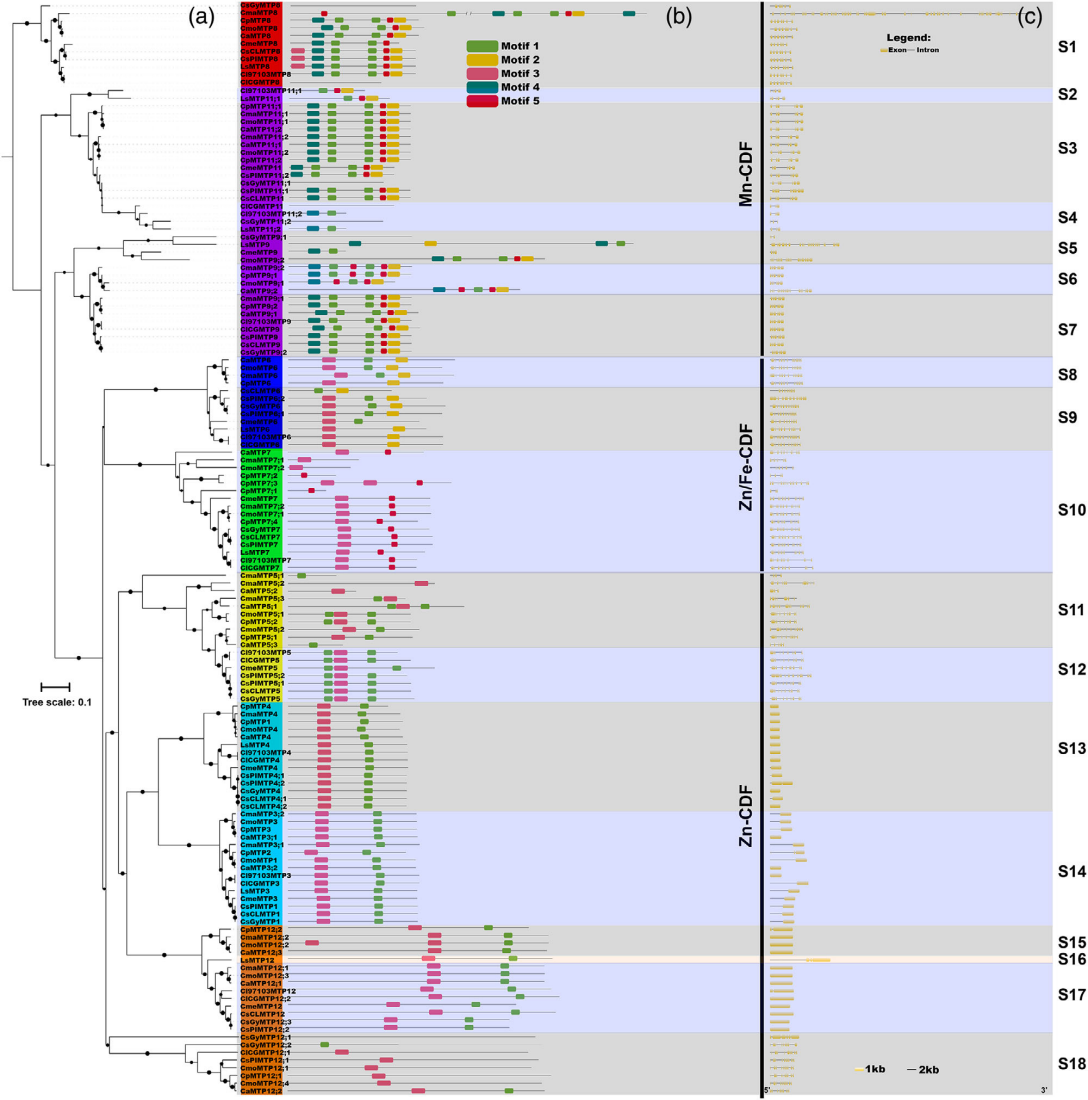
Distribution of the conserved motif and gene structure of metal‐tolerance protein (MTP) genes in Cucurbitaceae species. (A) Phylogenetic relationship and conserved motifs of MTP genes. Motifs 1 and 3 were related to the cation efflux family (Cation_efflux; PF01545), motif 2 was associated with the zinc transporter dimerization domain (ZT_dimer; PF16916), and motifs 4 and 5 did not relate to any motif. (B) Introns and exons are indicated by the yellow box and thin line

Protein motifs are highly conserved amino acid residues and may have functional and/or structural roles in active proteins (Vatansever et al., 2017). Five main conserved motifs were identified in all MTP*s* using MEME software (Figure 2). Approximate locations of five identified motifs were specified with different colored rectangles. The MTPs in each of the seven groups (i.e., G1, G5, G6, G7, G8, G9, and G12) had similar conserved motifs, but they were different between groups. All MTPs in the Mn‐MTP group had Motifs 1, 2, 4, and 5, except *Cucumis melo*, which only contained Motifs 1, 2, and 5. In the Mn‐MTP group, Motifs 1 and 3 were found in *Citrullus lanatus*, *Cucumis melo*, *Cucurbita*, and *Cucumis sativus*, and Motifs 3 and 5 were identified in *Lagenaria siceraria*. In the Mn‐MTP group, Motifs 2, 3, and 5 were identified in *Citrullus lanatus* and *Lagenaria siceraria*. Motifs 1, 2, 3, and 5 were found in *Cucumis sativus* and *Cucurbita*, whereas Motifs 1, 3, and 5 were identified in *Cucumis melo*. Motifs 1 (NVQSHFLHVJADTJGSVGVLLAGFILWFK) and 3 (DAAHLLSDVAAFAISLFALWASRWEADPQHSYGYGRLEILGALVS) belong to the cation efflux family (Cation_efflux; PF01545). Members of this family are integral membrane proteins that increase tolerance to divalent metal ions, such as Cd, Zn, and Co. These proteins are thought to be efflux pumps that remove these ions from cells. Motifs 1 and 3 were detected in almost all MTPs in the eight species. Moreover, Motif 2 comprised with residues NHHPEIKHIDTVRAYTFGVHYFVE was associated with the Zn transporter dimerization domain (ZT_dimer; PF16916), which was detected in the members of G6 (Zn/Fe‐CDF), G8 (Mn‐CDF), and G9 (Mn‐CDF). However, whether these domains were correlated with the functions of these MTPs is unknown. Motifs 4 (PERYVAEYYZRQVEMLKGFNEVDSLNELGYVPGMSTEEREK) and 5 (ENVNSLVGRSAPPEYLQKLTY) did not relate to any function of known motifs (Figure 2).

Exon‐intron structural diversity often plays a key role in the evolution of gene families and can provide additional evidence to support phylogenetic groupings (Zhu et al., 2020). To provide insights into the structure analysis of each MTP gene, exon‐intron structure analysis was performed. The difference in MTPs was significant. The number of exons varied among the members of the MTP family. As shown in Figure 2, the number of introns in MTPs ranged from 1 to 63. Generally, genes that clustered together had a similar gene structure (e.g., number and length of exons). For example, 142 MTPs were further divided into S1 (subgroup 1) and S18 based on the phylogenetic tree. Subgroups 13, S14, and S15 comprised one to three exons and introns. All S7 members contained 6 exons, and the S8 members contained 12–13 exons and 12 introns. Moreover, of the 13 S3 members, 11 comprise the same member of introns and exons. Overall, MTPs showed variations in intron position and length. However, their functions require further verification. This diversity facilitated the evolution of multigene families. These results suggest that exons are lost and gained during the evolution of the Cucurbitaceae MTPs, which may contribute to the functional diversity of the entire family of MTPs (Figure 2).

### 3.4 Synteny analysis of MTP genes

**FIGURE 3:**
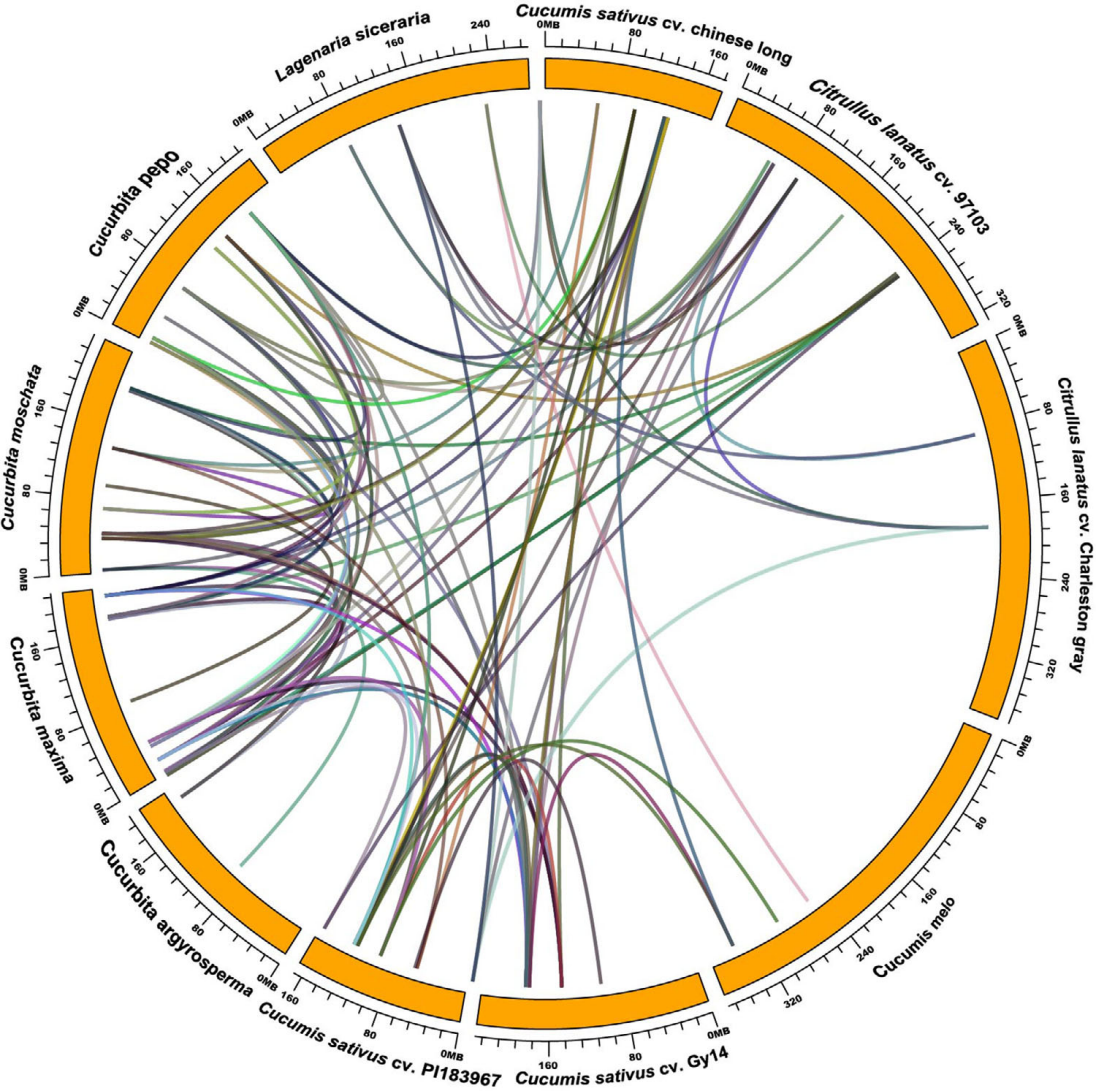
Synteny of metal‐tolerance protein (MTP) genes among Cucurbitaceae species. The orange box represents the sequence length of chromosomes in Mb. The lines with different colors indicate the syntenic relationship among MTP regions. Detailed synteny information for each two species is shown in Supplemental Figure S2

Segmental duplications refer to DNA sequences with an identity rate usually >90%, ranging from 1 to 400 kb in length and occurring on multiple sites within the genome (Ramsey & Schemske, 1998). Segmental duplications occur on different chromosomes, in contrast to tandem duplications, which occur on the same chromosome (Cannon et al., 2004). To investigate the evolutionary history and relationships of MTPs among different Cucurbitaceae species, a synteny analysis of MTPs was performed. A synteny map was constructed using orthologous gene pairs of the MTP genes among *Cucumis sativus*, *Citrullus lanatus*, *Cucumis melo*, *Cucurbita*, and *Lagenaria siceraria* (Figure 3). As shown in Figure 3, 98 pairs of MTP segmental‐like duplication events were found (Supplemental Table S3 and Supplemental Figure S2). Based on this analysis, two, four, 11, 22, six, and 12 collinear gene pairs were identified between *Cucumis sativus* and *Lagenaria siceraria*, *Cucumis sativus* and *Cucumis melo*, *Cucumis sativus* and *Citrullus lanatus*, *Cucumis sativus* and Cucurbita, *Citrullus lanatus* and *Lagenaria siceraria*, and *Citrullus lanatus* and *Cucurbita*, respectively. Among the same species, 12 collinear gene pairs were identified between CsGyMTP, CsPIMTP, and CsCLMTP. Similarly, 12 collinear gene pairs were identified between ClCGMTP and Cl97103MTP, and 25 collinear gene pairs were identified between CmaMTP, CmoMTP, and CpMTP (Supplemental Table S3 and Supplemental Figure S2). These results showed that some MTPs may have been generated by gene duplication, and segmental duplication events could have played a major driving force for MTP evolution.

The Ka/Ks values are widely used to represent gene selection pressure and the rate of evolution (Fang et al., 2020). The Ka/Ks ratio was calculated for 98 gene pairs (Supplemental Table S4). Generally, Ka/Ks > 1 indicates positive selection with accelerated evolution, Ka/Ks < 1 suggests purifying selection with a functional constraint, and Ka/Ks = 1 represents that the genes are drifting naturally. In this study, the Ka/Ks ratio of all MTP gene pairs was less than 1, suggesting that MTP genes are mainly under purifying/negative selection (Supplemental Table S5).

### 3.5 Expression profiling of Cucurbitaceae MTP genes in different developmental stages and under different treatments

**FIGURE 4:**
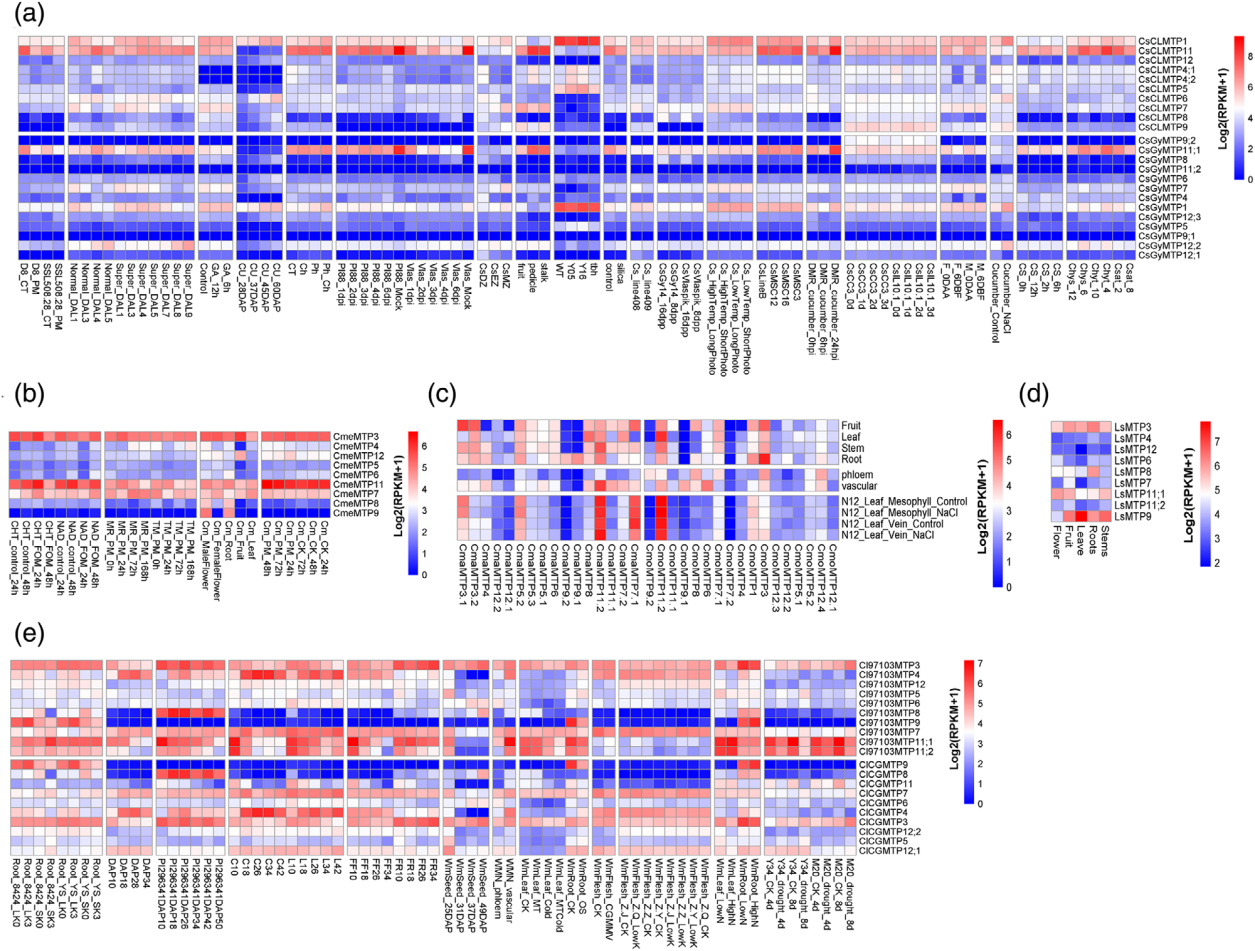
Expression pattern profiling of Cucurbitaceae metal‐tolerance protein (MTP) genes. (A) *Cucumis sativus*, (B) *Cucumis melo*, (C) *Cucurbita*, (D) *Lagenaria siceraria*, and (E) *Citrullus lanatus*. The log2(FPKM+1) transformed values of the dataset were used to perform expression analysis. Detailed information for each treatment is listed in Supplemental Table S6

The response of MTPs to heavy metal substrates has been indicated to be complicated and involved in multilayered regulatory mechanisms (Gao et al., 2020). To compare the expression patterns and reveal the possible function of Cucurbitaceae MTPs, the expression levels of MTPs in different tissues during different developmental stages and under various growth conditions were analyzed by the available RNA‐seq transcriptomic data. Based on the log2‐transformed FPKM values of the dataset, the different expression patterns of Cucurbitaceae were demonstrated in the heatmap (Figure 4). The expression of MTP genes varied among different species, as well as among different cultivars in the same species. In *Cucumis sativus* Chinese Long and Gy14, both MTP11 and MTP1 showed generally higher expression levels regardless of tissue type, development stage, or treatment. The expression levels of *MTP12;2* were generally higher in *C*. *sativus* Gy14, but not in Chinese Long (Figure 4A). In *C*. *melo*, *CmeMTP3*, *CmeMTP11*, and *CmeMTP7* were generally highly expressed. *CmeMTP7* and *CmeMTP8* were lowly expressed in different tissues, except in the roots and male flowers (Figure 4B). Furthermore, pathogen infection, including powdery mildew, *Podosphaera xanthii*, and *Fusarium oxysporum* f.sp. melonis Snyd. & Hans race 1.2, had little effect on the expression patterns of most CmeMTPs (Figure 4B). As shown in Figure 4B, in *Cucurbita*, MTP genes belonging to *Cucurbita maxima* (namely, *CmaMTP7;1*, *CmaMTP11;2*, *CmaMTP5;2* and *Cma3MTP;1*) and MTP genes belonging to *Cucurbita moschata* (namely, *CmoMTP11;2*, *CmoMTP1*, and *CmoMTP3*) showed higher expression levels in different tissues, including fruit, leaves (vein, mesophyll, vascular, and mesophyll), stem, and roots. *CmaMTP12;1*, *CmaMTP12;2*, *CmoMTP12;1*, and *CmoMTP12;2* showed relatively lower expression levels in all tissues (Figure 4C). *CmoMTP7;2* was lowly expressed in all tissues, while the expression levels of *CmaMTP7;2* were relatively higher in different tissues. Moreover, the expression levels of most *CmaMTP* and *CmoMTP* genes were not affected by NaCl treatment (Figure 4C). In *Lagenaria siceraria*, generally, *LsMTP3*, *LsMTP11;1*, and *LsMTP9* showed higher expression levels in different tissues (stem, root, leaf, fruit, and flower) than other *LsMTPs*. *LsMTP8* was more highly expressed in the roots than in other tissues (Figure 4D). In *Cucurbita argyrosperma*, *CaMTP3*, *CaMTP4*, and *CaMTP7* were generally higher expressed in both *Citrullus lanatus* 97103 and Charleston Gray. *Cl97103MTP11;1* and *Cl97103MTP11;2* showed higher expression levels in different tissues such as roots, leaves, fruit flesh, and fruit rind but not in the seed (Figure 4E). Moreover, drought stress slightly altered the expression levels of *Cl97103MTP* in different tissues but exerted little effect on the expression levels of *ClCGMTP* (Figure 4E).

Taken together, in each of these sequenced Cucurbitaceae species, MTP genes were constitutively expressed among different tissues and development stages. For example, among those MTPs identified in Cucurbitaceae species, *MTP11* showed higher expression levels in all Cucurbitaceae species and different cultivars, except for *ClCGMTP11*, suggesting the important role of MTP11 in the growth and development of Cucurbitaceae plants. The expression patterns of these Cucurbitaceae MTPs were not significantly altered by NaCl, drought, or pathogen infection (e.g., powdery mildew, *P*. *xanthii* and *F*. *oxysporum*). Further molecular and physiological study is required to characterize the stress response patterns under more stress conditions, especially heavy metal stress.

### 3.6 Expression patterns of CsMTPs under heavy metal toxicity

**FIGURE 5:**
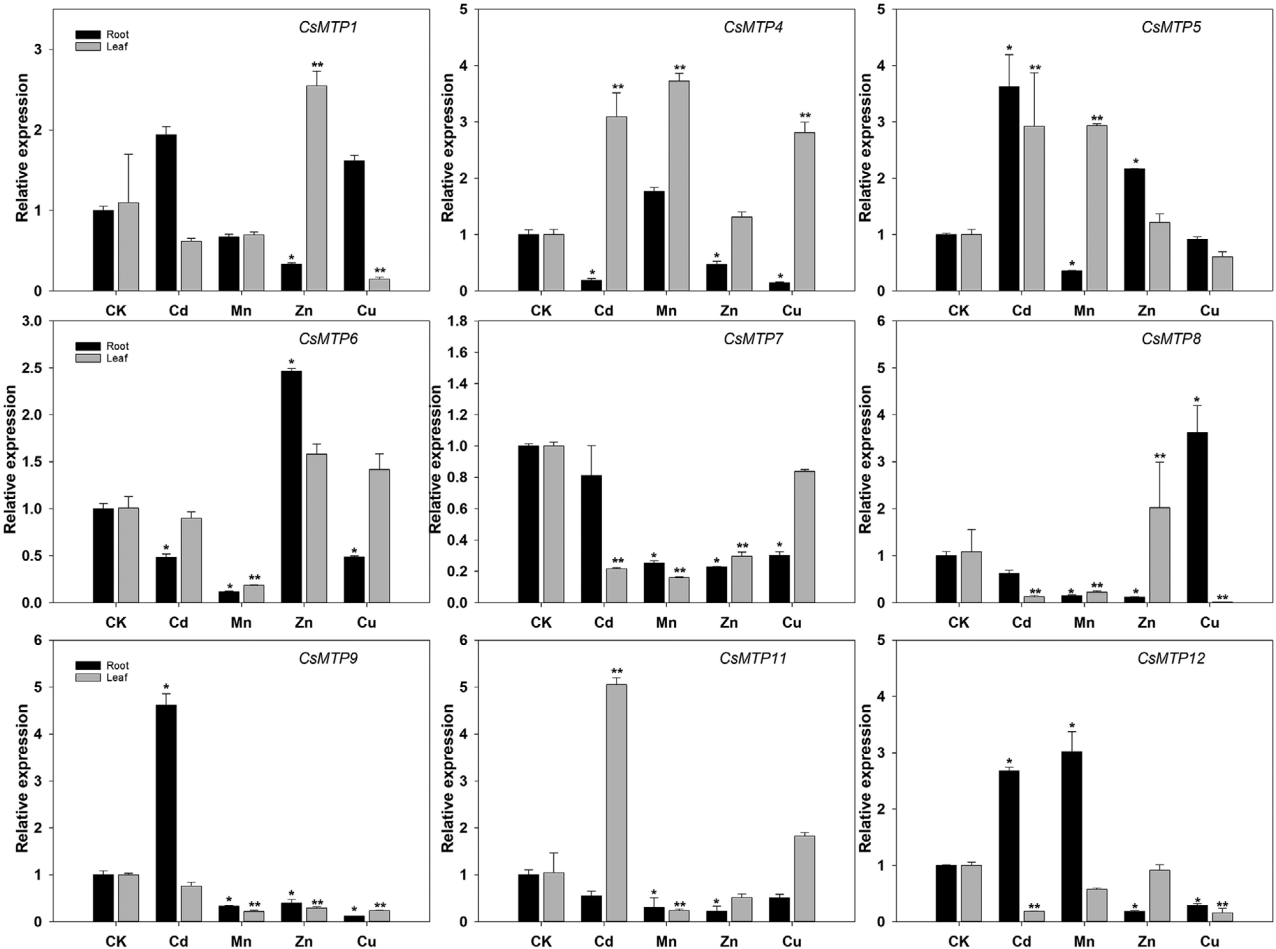
Relative expression levels of cucumber CsMTP genes under Cd, Mn, Zn, and Cu toxicity in roots and leaves. Quantitative polymerase chain reaction was used to analyze the relative expression levels of nine CsMTP genes in the roots and leaves under excess Zn, Mn, Cu, and Cd treatment for 3 d. * and ** indicate significant differences between the treatment samples and the corresponding control samples in roots and leaves

Members of plant MTPs have been reported to serve an important role in metal homeostasis and tolerance, including Zn, Cd, Co, Ni, Fe, and Mn (Li et al., 2018; Zhang & Liu, 2017). Thus, these transporters can be potentially used in the phytoremediation of heavy metal‐polluted soils. To determine the responsive patterns of cucumber CsMTP genes, root and leaf tissues subjected to Cd, Mn, Zn, and Cu toxicity for 3 d were collected to perform qPCR. A log_2_(fold‐change) > 1 was considered significantly upregulated under these treatments (Fu et al., 2017). The results showed that the expression of most CsMTP genes could be induced by different metals. For example, the expression levels of *CsMTP4*, *CsMTP5*, and *CsMTP11* were significantly induced by Cd treatment. *CsMTP4* and *CsMTP5* were also upregulated by Mn treatment in the leaves. *CsMTP8* and *CsMTP1* were significantly induced by Zn treatment in the roots. Furthermore, CsMTP genes could be induced by heavy metals that are not potential substrates for the MTP genes. For instance, under Cu stress, most of the *CsMTP* genes were significantly or slightly downregulated, except for *CsMTP4*, *CsMTP5*, and *CsMTP11*, which were significantly (*CsMTP4*) or slightly upregulated (*CsMTP5* and *CsMTP11*). In the roots, *CsMTP8*, *CsMTP6*, and *CsMTP9* were upregulated by Cu, Zn, and Cd treatment, respectively. Similar findings were also reported in poplar (*Populus trichocarpa* Torr. & A. Gray ex Hook) (Gao et al., 2020) and sweet orange (Fu et al., 2017) (Figure 5). The expression levels of *CsMTP1* were greatly induced by Cd and Zn treatment, and the expression levels of *CsMTP12* were largely increased by Cd and Mn stress. These results demonstrated that CsMTP genes showed different expression patterns under different metal toxicities. Generally, Cd treatment induced more CsMTP genes to be increased in both roots and leaves than Cu, Zn, and Mn treatments. Under Zn treatment, more CsMTP genes showed increased expression levels in leaves than in roots. Moreover, MTP genes may not respond to their transport substrates. For example, in the leaves, Zn‐CDF genes CsMTP5 and CsMTP5 were not significantly induced by Zn treatment for 3 d, which may be due to the Zn treatment concentrations used. To build a more comprehensive basis to decipher the physiological functions of MTPs in Cucurbitaceae plants, organ‐level expression of MTPs in a time course and in response to more heavy metal stressors should be further analyzed.

In summary, thus far, MTPs have been investigated in some plant species, such as Arabidopsis, wheat, sweet orange, poplar, and turnip, while no systematic analysis has been conducted in Cucurbitaceae plants. In this study, 142 MTP genes were identified from 13 accessible genomes belonging to eight Cucurbitaceae species, which were grouped into Mn‐CDF, Zn‐CDF, and Zn/Fe‐CDF, and their phylogenetic relationships, gene structures, chromosome distributions, conserved domains, and motifs were analyzed. Furthermore, previously released RNA‐seq data revealed the diverse expression patterns of MTP genes in five Cucurbitaceae species across different tissues and developmental stages. The qPCR analysis demonstrated the expression profiles of all of the cucumber MTP genes in response to different heavy metal stressors (Cd, Mn, Zn, and Cu), and the expression levels of all CsMTP members were induced by at least one metal ion. The present study provides essential insights into Cucurbitaceae MTP genes and lays the foundation for more in‐depth experimental exploration of the exact functions of these MTP genes.

## AUTHOR CONTRIBUTIONS

Xinchen Jiang: Investigation; Writing‐original draft. Junliang Yin: Data curation; Investigation; Resources; Writing‐original draft; Writing‐review & editing. Lixin Wang: Formal analysis; Writing‐original draft. Keyong Xi: Investigation. Xiongmeng Zhu: Investigation. Gang Li: Investigation. Yongxing Zhu: Conceptualization; Data curation; Funding acquisition; Investigation; Methodology; Project administration; Supervision; Writing‐original draft; Writing‐review & editing.Yiqing Liu: Project administration; Resources; Writing‐review & editing.

## CONFLICT OF INTEREST

The authors declare that they have no conflict of interests.

## Supporting information

Table S1. Primers of the CsMTP genes and reference genes.
Table S2. Synteny statistics of MTP genes among different Cucurbitaceae species.
Table S3. Duplication events in Cucurbitaceae species.
Table S4. Detailed information for duplication.
Table S5. Synonymous and non‐synonymous substitution rates for duplication events.
Table S6. Detailed information for RNA‐seq treatments.

Figure S1. Phylogenetic tree of MTP proteins from Cucurbitaceae species and Arabidopsis, maize, and rice MTPs.
Figure S2. Synteny of MTP genes between different Cucurbitaceae species.

File S1. Multiple sequence alignment of MTP proteins in different Cucurbitaceae species.

## Data Availability

The genome data and sequences and expression profiles of MTP genes used in the current study are available in the Cucurbit Genomics Database (http://cucurbitgenomics.org/search/genome/2). All data generated or analyzed during this study are included in this published article and its Supplementary files.

## References

[tpg220167-bib-0001] Bailey, T. L. , Boden, M. , Buske, F. A. , Frith, M. , Gran, C. E. , Clementi, L. , Ren, J. , Li, W. W. , & Noble, W. S. (2009). MEME SUITE: Tools for motif discovery and searching. Nucleic Acids Research, 37, W202–W208. 10.1093/nar/gkp335 19458158 PMC2703892

[tpg220167-bib-0002] Bhat, J. A. , Shivaraj, S. M. , Singh, P. , Navadagi, D. B. , Tripathi, D. K. , Dash, P. K. , Solanke, A. U. , Sonah, H. , & Deshmukh, R. (2019). Role of silicon in mitigation of heavy metal stresses in crop plants. Plants, 8, 71–91. 10.3390/plants8030071 30901942 PMC6473438

[tpg220167-bib-0003] Cannon, S. B. , Mitra, A. , Baumgarten, A. , Young, N. D. , & May, G. (2004). The roles of segmental and tandem gene duplication in the evolution of large gene families in *Arabidopsis thaliana* . BMC Plant Biology, 4, 10–31. 10.1186/1471-2229-4-10 15171794 PMC446195

[tpg220167-bib-0004] Chen, Z. , Fujii, Y. , Yamaji, N. , Masuda, S. , Takemoto, Y. , Kamiya, T. , Yusuyin, Y. , Iwasaki, K. , Kato, S. , Maeshima, M. , Ma, J. , & Ueno, D. (2013). Mn tolerance in rice is mediated by MTP8. 1, a member of the cation diffusion facilitator family. Journal of Experimental Botany, 64, 4375–4387. 10.1093/jxb/ert243 23963678 PMC3808320

[tpg220167-bib-0005] Chu, H. H. , Car, S. , Socha, A. L. , Hindt, M. N. , Punshon, T. , & Guerinot, M. L. (2017). The Arabidopsis MTP8 transporter determines the localization of manganese and iron in seeds. Scientific Reports, 7(1), 11024. 10.1038/s41598-017-11250-9 28887568 PMC5591227

[tpg220167-bib-0006] Fang, Z. , Jiang, W. , He, Y. , Ma, D. , Liu, Y. , Wang, S. , Zhang, Y. , & Yin, J. (2020). Genome‐wide identification, structure characterization, and expression profiling of Dof transcription factor gene family in wheat (*Triticum aestivum* L.). Agronomy, 10, 294. 10.3390/agronomy10020294

[tpg220167-bib-0007] Feng, Y. , Ma, L. Y. , Wang, Q. , Wu, Y. J. , Huang, L. K. , Zhou, Q. Y. , & Yang, X. E. (2018). Heavy‐metal pollution and safety production technologies of soil‐vegetable crop systems in China. Journal of Agro⁃Environment Science, 37, 2359–2370 (In Chinese with English abstract). 10.11654/jaes.2018-0787

[tpg220167-bib-0008] Fu, X. , Tong, Y. , Zhou, X. , Ling, L. , Chun, C. , Cao, L. , Zeng, M. , & Peng, L. (2017). Genome‐wide identification of sweet orange (*Citrus sinensis*) metal tolerance proteins and analysis of their expression patterns under zinc, manganese, copper, and cadmium toxicity. Gene, 629, 1–8. 10.1016/j.gene.2017.07.072 28760553

[tpg220167-bib-0009] Fujiwara, T. , Kawach, M. , Sato, Y. , Mori, H. , Kutsuna, N. , Hasezawa, S. , & Maeshima, M. (2015). A high molecular mass zinc transporter MTP 12 forms a functional heteromeric complex with MTP 5 in the Golgi in *Arabidopsis thaliana* . FEBS Journal, 282, 1965–1979. 10.1111/febs.13252 25732056

[tpg220167-bib-0010] Gao, Y. , Yang, F. , Liu, J. , Xie, W. , & Yao, Y. (2020). Genome‐wide identification of metal tolerance protein genes in populus trichocarpa and their roles in response to various heavy metal stresses. International Journal of Molecular Sciences, 21(5), 1680. 10.3390/ijms21051680 32121430 PMC7084629

[tpg220167-bib-0011] Ghori, N. H. , Ghori, T. , Hayat, M. Q. , Imadi, S. R. , Gul, A. , Altay, V. , & Ozturk, M. (2019). Heavy metal stress and responses in plants. International Journal of Environmental Science and Technology volume, 16, 1807–1828. 10.1007/s13762-019-02215-8

[tpg220167-bib-0012] Guo, A. Y. , Zhu, Q. H. , Chen, X. , & Luo, J. C. (2007). GSDS: A gene structure display server. Hereditas, 29, 1023–1026. 10.1360/yc-007-1023 17681935

[tpg220167-bib-0013] Gustin, J. L. , Zanis, M. J. , & Salt, D. E. (2011). Structure and evolution of the plant cation diffusion facilitator family of ion transporters. BMC Evolutionary Biology, volume, 11, 76–88. 10.1186/1471-2148-11-76 21435223 PMC3073911

[tpg220167-bib-0014] Haney, C. J. , Grass, G. , Franke, S. , & Rensing, C. (2005). New developments in the understanding of the cation diffusion facilitator family. Journal of Industrial Microbiology and Biotechnology, 32, 215–226. 10.1007/s10295-005-0224-3 15889311

[tpg220167-bib-0015] Hoagland, D. , & Arnon, D . (1950) The water culture method for growing plants without soil. Circular. California Agricultural Experiment Station, 347, 1–32.

[tpg220167-bib-0016] Lang, M. , Hao, M. , Fan, Q. , Wang, W. , Mo, S. , Zhao, W. , & Zhou, J. (2011). Functional characterization of *BJCET3* and *BJCET4*, two new cation‐efflux transporters from *Brassica juncea* L. Journal of Experimental Botany, 62, 4467–4480. 10.1093/jxb/err137 21652531 PMC3170545

[tpg220167-bib-0017] Li, X. , Wu, Y. , Li, B. , He, W. , Yang, Y. , & Yang, Y. (2018). Genome‐wide identification and expression analysis of the cation diffusion facilitator gene family in turnip under diverse metal ion stresses. Frontiers in Genetics, 9, 103–115. 10.3389/fgene.2018.00103 29670641 PMC5893799

[tpg220167-bib-0018] Librado, P. , & Rozas, J. (2009). DnaSP v5: A software for comprehensive analysis of DNA polymorphism data. Bioinformatics, 25, 1451–1452. 10.1093/bioinformatics/btp187 19346325

[tpg220167-bib-0019] Maleki, M. , Ghorbanpour, M. , & Kariman, K. (2017). Physiological and antioxidative responses of medicinal plants exposed to heavy metals stress. Plant Gene, 11, 247–254. 10.1016/j.plgene.2017.04.006

[tpg220167-bib-0020] Migocka, M. , Kosieradzka, A. , Papierniak, A. , Maciaszczyk‐Dziubinska, E. , & Filleur, S. (2015). Two metal‐tolerance proteins, MTP1 and MTP4, are involved in Zn homeostasis and Cd sequestration in cucumber cells. Journal of Experimental Botany, 66, 1001–1015. 10.1093/jxb/eru459 25422498

[tpg220167-bib-0021] Migocka, M. , Papierniak, A. , Kosieradzka, A. , Posyniak, E. , Maciaszczyk‐Dziubinska, E. , Biskup, R. , Garbiec, A. , & Marchewka, T. (2015). Cucumber metal tolerance protein CSMT P9 is a plasma membrane H^+^‐coupled antiporter involved in the Mn^2+^ and Cd^2+^ efflux from root cells. The Plant Journal, 84, 1045–1058. 10.1111/tpj.13056 26485215

[tpg220167-bib-0022] Migocka, M. , Papierniak, A. , Maciaszczyk‐Dziubinska, E. , Pondzik, P. , Posyniak, E. , Garbiec, A. , & Filleur, S. (2014). Cucumber metal transport protein MTP8 confers increased tolerance to manganese when expressed in yeast and *Arabidopsis thaliana* . Journal of Experimental Botany, 65, 5367–5384. 10.1093/jxb/eru295 25039075 PMC4400539

[tpg220167-bib-0023] Montanini, B. , Blaudez, D. , Jeandroz, S. , Sanders, D. , & Chalot, M. (2007). Phylogenetic and functional analysis of the Cation Diffusion Facilitator (CDF) family: Improved signature and prediction of substrate specificity. BMC Genomics, 8, 107–122. 10.1186/1471-2164-8-107 17448255 PMC1868760

[tpg220167-bib-0024] Peiter, E. , Montanini, B. , Gobert, A. , Pedas, P. , Husted, S. , Maathuis, F. J. M. , Blaudez, D. , Chalot, M. , & Sanders, D. (2007). A secretory pathway‐localized cation diffusion facilitator confers plant manganese tolerance. Proceedings of the National Academy of Sciences of the United States of America, 104, 8532–8537. 10.1073/pnas.0609507104 17494768 PMC1895984

[tpg220167-bib-0025] Rajasree, R. S. , Sibi, P. I. , Francis, F. , & William, H. (2016). Phytochemicals of Cucurbitaceae family: A review. International Journal of Pharmacognosy and Phytochemical Research, 8, 113–123.

[tpg220167-bib-0026] Ramsey, J. , & Schemske, D. W. (1998). Pathways, mechanisms, and rates of polyploid formation in flowering plants. Annual Review of Ecology and Systematics, 29, 467–501. 10.1146/annurev.ecolsys.29.1.467

[tpg220167-bib-0027] Renner, S. S. , & Schaefer, H. (2016). Phylogeny and evolution of the Cucurbitaceae genetics and genomics of Cucurbitaceae. Genetics and Genomics of Cucurbitaceae, 20, 13–23. 10.1007/7397_2016_14

[tpg220167-bib-0028] Ricachenevsky, F. K. , Menguer, P. K. , Sperotto, R. A. , Williams, L. E. , & Fett, J. P. (2013). Roles of plant metal tolerance proteins (MTP) in metal storage and potential use in biofortification strategies. Frontiers in Plant Science, 4, 144–159. 10.3389/fpls.2013.00144 23717323 PMC3653063

[tpg220167-bib-0029] Tamura, K. , Stecher, G. , Peterson, D. , Filipski, A. , & Kumar, S. (2013). MEGA6: Molecular evolutionary genetics analysis version 6.0. Molecular Biology and Evolution, 30, 2725–2729. 10.1093/molbev/mst197 24132122 PMC3840312

[tpg220167-bib-0030] Vatansever, R. , Filiz, E. , & Eroglu, S. (2017). Genome‐wide exploration of metal tolerance protein (*MTP*) genes in common wheat (*Triticum aestivum*): Insights into metal homeostasis and biofortification. Biometals, 30, 217–235. 10.1007/s10534-017-9997-x 28150142

[tpg220167-bib-0031] Voorrips, R. E. (2002). MapChart: Software for the graphical presentation of linkage maps and QTLs. Journal of Heredity, 93(1), 77–78. 10.1093/jhered/93.1.77 12011185

[tpg220167-bib-0032] Wang, Y. , Tang, H. , DeBarry, J. D. , Tan, X. , Li, J. , Wang, X. , Lee, T. , Jin, H. , Marler, B. , Guo, H. , Kissinger, J. C. , & Paterson, A. H. (2012). MCScanX: A toolkit for detection and evolutionary analysis of gene synteny and collinearity. Nucleic Acids Research, 40, e49. 10.1093/nar/gkr1293 22217600 PMC3326336

[tpg220167-bib-0033] Wu, J. W. , Mock, H. P. , Giehl, R. F. H. , Pitann, B. , & Muehling, K. H. (2019). Silicon decreases cadmium concentrations by modulating root endodermal suberin development in wheat plants. Journal of Hazardous Materials, 364, 581–590. 10.1016/j.jhazmat.2018.10.052 30388642

[tpg220167-bib-0034] Wu, J. W. , Shi, Y. , Zhu, Y. , Wang, Y. , & Gong, H. (2013). Mechanisms of enhanced heavy metal tolerance in plants by silicon: A review. Pedosphere, 23, 815–825. 10.1016/S1002-0160(13)60073-9

[tpg220167-bib-0035] Yang, S. L. , & Walters, T. W. (1992). Ethnobotany and the economic role of the Cucurbitaceae of China. Economic Botany, 46(4), 349–367. 10.1007/BF02866506

[tpg220167-bib-0036] Yin, J. L. , Liu, M. Y. , Ma, D. F. , Wu, J. W. , Li, S. L. , Zhu, Y. X. , & Han, B. (2018). Identification of circular RNAs and their targets during tomato fruit ripening. Postharvest Biology and Technology, 136, 90–98. 10.1016/j.postharvbio.2017.10.013

[tpg220167-bib-0037] Yin, J. , Wang, L. , Zhao, J. , Li, Y. , & Zhu, Y. (2020). Genome‐wide characterization of the C2H2 zinc‐finger genes in *Cucumis sativus* and functional analyses of four *CsZFPs* in response to stresses. BMC Plant Biology, 20, 359–380. 10.1186/s12870-020-02575-1 32727369 PMC7392682

[tpg220167-bib-0038] Yuan, L. , Yang, S. , Liu, B. , Zhang, M. , & Wu, K. (2012). Molecular characterization of a rice *metal tolerance protein*, *OsMTP1* . Plant Cell Reports, 31, 67–79. 10.1007/s00299-011-1140-9 21892614

[tpg220167-bib-0039] Zhu, Y. X. , Gong, H. J. , & Yin, J. L. (2019a). Role of silicon in mediating salt tolerance in plants: A review. Plants, 8(6), 147. 10.3390/plants8060147 31159197 PMC6630593

[tpg220167-bib-0040] Zhu, Y.‐X. , Jia, J.‐H. , Yang, L. , Xia, Y.‐C. , Zhang, H.‐L. , Jia, J.‐B. , Zhou, R. , Nie, P.‐Y. , Yin, J.‐L. , Ma, D.‐F. , & Liu, L.‐C. (2019b). Identification of cucumber circular RNAs responsive to salt stress. BMC Plant Biology, 19, 164. 10.1186/s12870-019-1712-3 31029105 PMC6486992

[tpg220167-bib-0041] Zhu, Y. , Jiang, X. , Zhang, J. , He, Y. , & Liu, Y. (2020). Silicon confers cucumber resistance to salinity stress through regulation of proline and cytokinins. Plant Physiology and Biochemistry, 156, 209–220. 10.1016/j.plaphy.2020.09.014 32977177

[tpg220167-bib-0042] Zhu, Y. , Yang, L. , Liu, N. , Yang, J. , Zhou, X. , Xia, Y. , He, Y. , He, Y. , Gong, H. , Ma, D. , & Yin, J. (2019c). Genome‐wide identification, structure characterization, and expression pattern profiling of aquaporin gene family in cucumber. BMC Plant Biology, 19, 345. 10.1186/s12870-019-1953-1 31390991 PMC6686268

[tpg220167-bib-0043] Zhang, M. , & Liu, B. (2017). Identification of a rice metal tolerance protein OsMTP11 as a manganese transporter. Plos One, 12, e174987. 10.1371/journal.pone.0174987 PMC5386239 28394944

[tpg220167-bib-0044] Zhang, X. , Li, Q. , Xu, W. , Zhao, H. , & Wei, C. (2020). Identification of MTP gene family in tea plant (*Camellia sinensis* L.) and characterization of CsMTP8.2 in manganese toxicity. Ecotoxicology and Environmental Safety, 202, 110904. 10.1016/j.ecoenv.2020.110904 32800239

