# Supplementary material for: Identification and evolutionary analysis of the metal‐tolerance protein family in eight Cucurbitaceae species
Source: Plant Genome. 2021 Nov 6;15(1):e20167. doi: 10.1002/tpg2.20167 (PMC12807381; doi:10.1002/tpg2.20167)
Supplement: Supplementary file 1 [file TPG2-15-e20167-s003.docx]

Figure S1. Phylogenetic tree of MTP proteins from Cucurbitaceae species and Arabidopsis, maize and rice MTPs. The tree was generated with MEGA7 software using the neighbor-joining method using a total of 12, 19, and 10 sequence from Arabidopsis, maize, and rice. The proteins were divided into three groups (Zn-MTPs, Fe/Zn-MTPs, and Mn-MTPs) and further divided into 7 groups of G1, G5, G6, G7, G8, G9, and G12 (light blue: G1;yellow: G5; dark blue: G6; green: G7; red: G8; purple: G9; orange: G12).

Figure S2. Synteny of MTP genes between different Cucurbitaceae species. (A) Synteny analyses of MTP genes between 3 species of *Cucumis sativus*; (B) Synteny analyses of MTP genes among 3 species of *Cucumis sativus* and 2 species of *Citrullus lanatus*; (C) Synteny analyses of MTP genes among different species of *Cucumis sativus* and *Cucumis melo*; (D) Synteny analyses of MTP genes among 3 species of *Cucumis sativus* and *Lagenaria siceraria*; (E) Synteny analyses of MTP genes among 3 species of *Cucumis sativus* and 4 species of *Cucurbita*; (F) Synteny analyses of MTP genes between 2 species of *Citrullus lanatus*; (G) Synteny analyses of MTP genes between 2 species of *Citrullus lanatus* and *Lagenaria siceraria*; (H) Synteny analyses of MTP genes between 2 species of *Citrullus lanatus* and 4 species *Cucurbita*; (I) Synteny analyses of MTP genes among 4 species *Cucurbita*.
